# Neuronal correlates of asocial behavior in a BTBR *T*^+^*Itpr3^tf^/J* mouse model of autism

**DOI:** 10.3389/fnbeh.2015.00199

**Published:** 2015-08-06

**Authors:** Ksenia Meyza, Tomasz Nikolaev, Kacper Kondrakiewicz, D. Caroline Blanchard, Robert J. Blanchard, Ewelina Knapska

**Affiliations:** ^1^Laboratory of Emotions’ Neurobiology, Department of Neurophysiology, Nencki Institute of Experimental Biology PASWarsaw, Poland; ^2^Department of Psychology, University of Hawaii at ManoaHonolulu, HI, USA; ^3^Pacific Biosciences Research Center, University of Hawaii at ManoaHonolulu, HI, USA

**Keywords:** autism, mouse model, empathy, BTBR, c-Fos

## Abstract

Autism spectrum disorder (ASD) is a neurodevelopmental disorder characterized, in part, by an inability to adequately respond to social cues. Patients diagnosed with ASD are often devoid of empathy and impaired in understanding other people’s emotional perspective. The neuronal correlates of this impairment are not fully understood. Replicating such a behavioral phenotype in a mouse model of autism would allow us insight into the neuronal background of the problem. Here we tested BTBR *T^+^Itpr3^tf^/J* (BTBR) and c57BL/6J (B6) mice in two behavioral paradigms: the Transfer of Emotional Information test and the Social Proximity test. In both tests BTBR mice displayed asocial behavior. We analyzed c-Fos protein expression in several brain regions after each of these tests, and found that, unlike B6 mice, BTBR mice react to a stressed cagemate exposure in the Transfer of Emotional Information test with no increase of c-Fos expression in either the prefrontal cortex or the amygdala. However, after Social Proximity exposure we observed a strong increase in c-Fos expression in the CA3 field of the hippocampus and two hypothalamic regions of BTBR brains. This response was accompanied by a strong activation of periaqueductal regions related to defensiveness, which suggests that BTBR mice find unavoidable social interaction highly aversive.

## Introduction

Autism spectrum disorder (ASD) is an increasingly common (Fombonne, 2003; Maenner and Durkin, 2010; Jo et al., 2015), behaviorally defined neurodevelopmental disorder diagnosed by two major clusters of symptoms: (1) deficits in social communication and social interaction; and (2) restricted, repetitive patterns of behavior (DSM-V 299.0, American Psychiatric Association, 2013). Despite many years of scientific interest, the molecular and neurobiological bases of the disorder are still far from clear. This is mainly due to the polygenic (over a hundred contributing genes) character of the disorder (Betancur, 2011; Geschwind, 2011). With only 10–15% of cases associated with monogenic disorders such as Fragile X mental retardation syndrome (FXS), Rett’s syndrome (RTT) or tuberous sclerosis complex (TSC), the search for biological markers of relevant behavioral impairments relies heavily on validation of idiopathic models of the disorder.

The BTBR *T*^+^*Itpr3^tf^/J* (BTBR) inbred strain of mice is the most studied mouse model of idiopathic ASD. It displays all core behavioral features of ASD (Blanchard et al., 2012; Meyza et al., 2013). As underlined by the current ASD criteria (DSM-V 299.0, American Psychiatric Association, 2013), communication is crucial for development of the normo-social behavioral repertoire. BTBR mice display aberrant scent marking behavior (Roullet et al., 2011; Wöhr et al., 2011) as well as abnormal ultrasonic vocalization patterns both as pups (Scattoni et al., 2008) and adults (Scattoni et al., 2011, 2013; Yang et al., 2013). Recently, with the use of maternal separation induced vocalizations, it was shown that although BTBR pups respond to the scent of home cage bedding, they display impaired adjustment of the acoustic features of their calls to the changed environment (Wöhr, 2015). This is a very important finding, as it parallels an impairment seen in ASD patients which is difficult to model in mice. ASD patients are unable to appropriately adjust their behavior in response to social stimuli provided by other people, a feature described as empathy impairment (Cassidy et al., 2015).

Empathy is considered to be a multilayered phenomenon. In its simplest form it is characterized as a capacity to be affected by and/or to share the emotional state of another being, a phenomenon also known as emotional contagion (de Waal, 2008). Tuning one’s emotional state to that of another increases the probability of similar behavior, which thereby allows rapid adaptation to environmental challenges (Hatfield et al., 2009). The past few years have brought a number of observations suggesting that emotional contagion exists in rodents. Several studies showed that rats and mice are able to socially share states of fear (Knapska et al., 2006; Bredy and Barad, 2008; Chen et al., 2009; Jeon et al., 2010; Panksepp and Lahvis, 2011; Panksepp and Panksepp, 2013).

To address whether an emotional contagion phenomenon could be used for validation of mouse models of ASD we employed a model of between-subject Transfer of Emotional Information designed in our laboratory for testing emotional contagion in rats (Knapska et al., 2006). With this model we were able to show the neuronal correlates of the contagion, i.e., strong activation of the central and basolateral amygdala and the prefrontal cortex (Knapska et al., 2006; Mikosz et al., in press) of the Observer rat interacting with a previously stressed Demonstrator. Exposure to the same protocol was also found to improve subsequent learning in rats (Knapska et al., 2009). It was therefore interesting to see whether similar brain circuits are involved in emotional contagion in mice. To do this we looked at the expression of c-Fos protein in the amygdala and prefrontal cortex of both the Demonstrator and Observer B6 and BTBR mice from pairs where the Demonstrator was either stressed or left undisturbed (non-stressed control, Experiment 1). Since the role of the ventral hippocampus in the modulation of social behavior has recently been emphasized (Felix-Ortiz and Tye, 2014) we included that region in the analysis.

To verify whether brief social interaction alone is capable of inducing c-Fos protein expression in similar brain regions to those affected by Transfer of Emotional Information, we tested B6 and BTBR mice in the Social Proximity test (Experiment 2). It was previously reported (Defensor et al., 2011) that BTBR mice avoid direct nose-to-nose/head contacts in this paradigm and that the behavior is most clearly expressed in B6-BTBR mixed pairs of animals. This type of behavior is thought to parallel gaze aversion observed in autistic patients. To ensure that the observed c-Fos protein expression pattern was evoked by social interactions we compared it to the patterns following exposure to an empty Novel Arena and the Home Cage. Since the avoidance of contact in BTBR mice (Defensor et al., 2011) was previously discussed in terms of defensive mechanisms, we extended the c-Fos protein expression analysis to several brain regions regulating defensive behaviors.

## Materials and Methods

### Subjects

A total of 39 BTBR *T*^+^*Itpr3^tf^/J* (BTBR) and 53 c57BL/6J (B6) young adult (3 months of age) males were used for the study. The animals were bred from the original breeding pairs purchased from Jacksons Laboratory (Bar Harbor, ME, USA) kept as colonies at either the animal facilities of the University of Hawaii at Manoa, USA or the animal facilities of the Faculty of Biology, University of Warsaw, Poland. The conditions were kept as close as possible at both breeding colonies, i.e., animals were bred and housed at L:D 12:12 with temperature of 21 ± 2°C and 70% humidity. The food (standard laboratory chow) and tap water were available *ad libitum*. The animals were housed in standard Plexiglas cages with up to six animals per cage until the onset of behavioral habituation.

### Behavioral Testing

All experimental procedures performed at the University of Hawaii at Manoa followed NIH guidelines and were approved by the Institutional Animal Care and Use Committee at the University of Hawaii, protocol # 09-786-2. The experimental procedures performed at the Nencki Institute of Experimental Biology, Warsaw, Poland were approved by the Local Ethical Committee and performed in accordance with the ethical standards of European directive no. 2010/63/UE and Polish regulations.

#### Experiment 1: Transfer of Emotional Information

**Figure 1:**
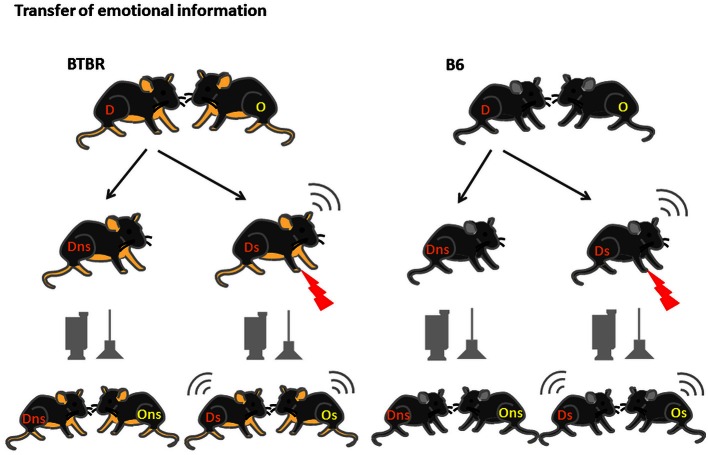
**Transfer of Emotional Information test—experimental design**. D—Demonstrators, O—Observers, are housed in fixed, same-strain pairs for at least 3 weeks prior to the onset of the test. After 2 weeks of habituation to being moved to the experimental room, handling by the experimenter and 10 min separation, on test day Demonstrators from the stressed group (D s) are exposed to 10 × 0.6 mA shocks an then reunited with Observers (O s). In the non-stressed group (D and O ns) the Demonstrators are placed in the conditioning chamber but no shocks are applied.

The animals bred and housed at the animal facilities of the Faculty of Biology, University of Warsaw, Poland (BTBR *n* = 24, B6 *n* = 28) were transferred to the Animal House of the Nencki Institute of Experimental Biology, Warsaw, Poland approximately 3 weeks before the onset of the experiment. The housing conditions were identical to those at the Faculty of Biology, University of Warsaw, Poland with one difference. Upon transfer the animals were separated into weight-matched pairs and housed in these pairs, in standard (35 × 17 × 13 cm) macrolon cages until the end of the experiment. After about a week of acclimatization, the habituation to the experimental room and handling by the experimenters started. During that time (10 days) the animals were transported to the experimental room and briefly separated (10 min) daily. By marking the tail of the animal taken away from the home cage we ensured that the same animal was removed from the home cage on every occasion. Later this animal will serve as a Demonstrator. The other animal (Observer) was left undisturbed in the home cage throughout the entire habituation. On the testing day the Demonstrator was placed in the fear conditioning apparatus (MED-Associates) and either left there undisturbed for 10 min or exposed to ten 0.6 mA footshocks. After the return of the Demonstrator to the home cage the behavior and ultrasonic vocalization of both animals (the Demonstrator and the Observer, Figure 1) were recorded for 10 min with the use of a digital camera hung above the home cage and an ultrasonic microphone connected to the UltraSoundGate device (Avisoft, Germany).

#### Experiment 2: Social Proximity

The animals bred at the University of Hawaii at Manoa, USA (BTBR *n* = 15 and B6 *n* = 15 + *n* = 10 of age and weight matched animals used as social interaction partners) were moved from the breeding facility to the experimental room 48 h prior to the onset of behavioral tests and placed in individual cages. On the day of the experiment, the animals were either taken directly from their home cages (control group, *n* = 5 BTBR and *n* = 5 B6) or subjected to one of the two behavioral tests: the solitary Novel Arena exposure test or Social Proximity test. The solitary Novel Arena test consisted of placing the animal alone for 10 min in a small novel arena, (7 × 14 × 30 cm transparent Plexiglas, *n* = 5 per strain). The Social Proximity test was a 10 min free interaction with an unfamiliar B6 mouse in the same small arena (*n* = 5 of each strain, as previously described in Defensor et al., 2011).

### Behavioral Data Analysis

The analysis of behavior digitally recorded during the Transfer of Emotional Information test, including number and duration of all contacts, nose-to-nose contacts, nose-to-tail contacts (according to parameters specified in Defensor et al., 2011) and following initiated by the Observer mouse (according to parameters specified in Pobbe et al., 2010) as well as the number and duration of digging episodes (performed by both the Demonstrator and the Observer) was scored using BehaView software written by dr Paweł Boguszewski from the Laboratory of the Limbic System, Nencki Institute of Experimental Biology, Warsaw, Poland.^1^

During the entire Transfer of Emotional Information test we recorded too few vocalizations from both B6 and BTBR pairs to allow quantitative analysis.

Behavior during the Social Proximity test was digitally recorded and afterwards offline scored using Observer 3.1 (Noldus, Netherlands) software for the total number and duration of Nose-to-Nose, Nose-to-Face, Nose-to-Tail Sniff, Crawl Under, Crawl Over, Allogrooming, Self-Grooming and Upright behaviors (according to parameters specified in Defensor et al., 2011).

### c-Fos Immunocytochemistry

Ninety minutes following the onset of the behavioral tests, the animals were killed with (>90 mg/kg) pentobarbital and perfused transcardially with ice-cold phosphate buffer saline (PBS, pH = 7.4) and then 4% paraformaldehyde in PBS (PFA, Sigma). The brains were removed and stored overnight in 4% PFA and subsequently flash frozen in isopentane (n-heptane, POCh) and kept at −80°C until the day of sectioning into 45 μm slices. The brain slices corresponding to: (a) medial prefrontal cortex, (AP + 1.70, Paxinos and Franklin, 2001), nucleus accumbens (AP + 1.18), hypothalamus (AP −0.94, −1.58), amygdala and dorsal hipppcampus (AP −1.58), premammilary bodies (AP −2.46), and periaqueductal gray (AP −4.36) for animals subjected to social interaction test; and (b) medial prefrontal cortex, (AP + 1.70), amygdala (AP −1.58), and ventral hippocampus (AP −3.08, Paxinos and Franklin, 2001) for animals that underwent transfer of emotional information, were chosen. The chosen slices were subjected to a free-float immunoreaction with the use of anti-c-Fos antibody (Santa Cruz sc-52, 1:1000, 48 h in 4°C), Vector, BA-1000 secondary antibody (1:400, 2 h at room temperature) and Vectastain ABC Kit (Vector PK 6100, 1 h at room temperature). The immunoreaction was developed with metal-enhanced DAB (Sigmafast DAB D0426–50SET) and Peroxidase (for about a minute) and stopped with three immediate rinses with PBS. The slices were then mounted on gel-coated slides and allowed to dry for 48 h, in room temperature. Subsequently they were dehydrated for 1 min in: Ethanol (70, 90, 96, 100%), Ethanol: Xylen: 50% 50% and twice in Xylen 100% and then closed with Entellan new (Merck) medium.

### Quantification of c-Fos Expression

The quantification of c-Fos immunostaining was performed with the use of ImageJ software (NIH). The borders of all regions of interest (ROIs) were delineated on the basis of neighboring, Nissl stained slices. For each slide or a set of structures (e.g., the whole hippocampus or amygdala) individual thresholds for recognition of c-Fos positive puncta were set manually. The neuronal activation is presented in arbitrary units ([a.u.], number of dots/area in pixels). c-Fos expression was assessed bilaterally on at least two sections relevant for a given structure by two independent viewers, blind to experimental conditions.

### Statistical Analysis

The behavioral parameters were tested for normal distribution and subsequently subjected to non-parametric one-way Kruskal-Wallis ANOVA (henceforth referred to as ANOVA). Within strain and between strain comparisons were made separately. Differences with *p* < 0.05 were considered significant.

The c-Fos data from the score averaged across two bilaterally assessed slices per ROI was tested, for each of the ROI separately, for normal distribution. Thereafter it was assessed with non-parametric one-way Kruskal-Wallis ANOVA with either strain or condition as independent variables. For the data obtained following the Transfer of Emotional Information Test, two sets of comparisons were made: non-stressed vs. stressed Demonstrator condition (in Demonstrators and Observers separately) and between Demonstrators and Observers in a given experimental group. For Social Proximity data, experimental groups exposed to Novel Arena and Social Proximity groups were compared with Home Cage controls and with one another. Results were considered significant with *p* < 0.05.

## Results

### Experiment 1: Transfer of Emotional Information

#### Behavioral Data

**Figure 2:**
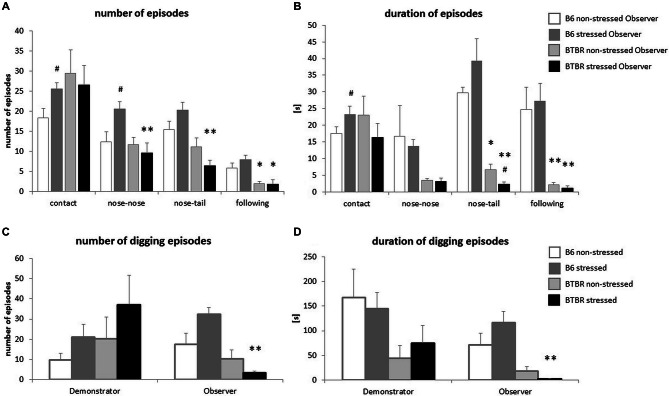
**Transfer of Emotional Information—unlike c57BL/6J (B6) mice, BTBR *T^+^Itpr3^tf^/J* (BTBR) mice do not display prosocial behaviors towards their stressed cagemates. (A)** The frequency of behaviors displayed by Observer mice during first 10 min of reunion with either non-stressed or stressed Demonstrators, **(B)** Duration of these behaviors, **(C)** Frequency of digging performed by both non-stressed and stressed Demonstrators and Observers, **(D)** Duration of these digging episodes. White bars represent non-stressed c57BL/6J (B6) mice, dark gray bars represent stressed B6 mice, light gray bars represent non-stressed BTBR *T^+^Itpr3^tf^/J* (BTBR) mice and black bars represent stressed BTBR mice. Values presented as mean. Error bars represent SEM. ^#^*p* < 0.05 for within strain comparisons, *p < 0.05, **p < 0.01, for between strain comparisons.

In response to a stressed cagemate the B6 Observer mice displayed an increase in the number and duration of social contacts (*p* < 0.05, Figures 2A,B), with special emphasis on the number of nose-to-nose contacts (*p* < 0.05, Figure 2A). The number of nose-to-tail contacts was also elevated, but the increase did not reach significance (*p* < 0.08). The number of nose-to-nose contacts made by B6 Observers exposed to a stressed cagemate was higher than that made by their BTBR counterparts (*p* < 0.01, Figure 2A) Similarly, B6 Observers exposed to a stressed cagemate made more and longer nose-to-tail contacts than the BTBR Observers in the same situation (*p* < 0.01, Figures 2A,B). Moreover, upon exposure to a stressed cagemate, BTBR mice inhibited rather than increased nose-to-tail contacts (*p* < 0.05, Figure 2B).

Another form of interaction, the following of the Demonstrator by the Observer, was also dependent on the strain and the emotional state of the cagemates. It was more prominent in B6 mice already in control conditions (*p* < 0.05 for the number of episodes and *p* < 0.01 for the duration of the episodes, Figures 2A,B). Exposure to a stressed cagemate did not change these parameters (*p* < 0.01 for either the number or duration of the episodes, Figures 2A,B).

Parallel to social behavior alterations, the differences in digging behavior were observed. BTBR Observers exposed to a stressed cagemate dug less and for shorter periods of time than B6 mice in the same conditions (*p* < 0.01, Figures 2C,D). B6 Observers exposed to a stressed Demonstrator dug slightly more often than when exposed to a non-stressed cagemate (*p* < 0.08), while no such effect was observed for BTBR mice.

#### c-Fos Protein Expression

Striking differences in the behavioral response to a stressed cagemate were paralleled by differences in c-Fos protein expression in the key structures responsible for regulation of emotions. The lack of behavioral response of BTBR Observers to a stressed cagemate was followed by a lack of increase in c-Fos protein expression in several brain regions. For the purpose of this experiment, the prefrontal cortex was divided into prelimbic and infralimbic areas. The amygdalar complex was divided into basal/basolateral, lateral, central lateral, central medial, medial and cortical nuclei. The ventral part of hippocampus was divided into the CA1 and CA3 fields and the dentate gyrus (DG).

##### Prefrontal cortex

**Figure 3:**
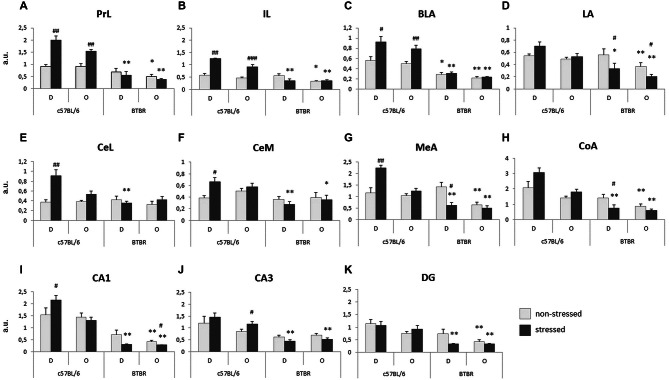
**Transfer of Emotional Information—unlike c57BL/6J (B6) mice, BTBR *T^+^Itpr3^tf^/J* (BTBR) mice do not display increase of c-Fos protein expression in response to direct or socially transmitted stress in: (A) prelimbic cortex, **(B)** infralimbic cortex (IL), **(C)** basal/basolateral nucleus (BLA) of the amygdala, **(D)** LA of the amygdala, **(E)** lateral part of the central nucleus of the amygdala, **(F)** medial part of the central nucleus of the amygdala, **(G) medial** nucleus of the amygdala, **(H)** cortical nucleus of the amygdala, **(I)** CA1 field of the ventral hippocampus, **(J)** CA3 field of the ventral hippocampus, **(K)** dentate gyrus (DG) of the ventral hippocampus**. D—Demonstrators, O—Observers. Light gray bars represent non-stressed pairs, dark gray bars represent stressed pairs. Values presented as mean. Error bars represent SEM. ^#^*p* < 0.05, ^##^*p* < 0.01, ^###^*p* < 0.001 for within strain comparisons, and *p < 0.05, **p < 0.01 for between strain comparisons.

The expression of c-Fos protein in the prelimbic cortex (PrL) was analyzed for between strain (B6 and BTBR) and between conditions (non-stressed vs. stressed Demonstrator in a given pair) separately. In B6 mice both the stressed Demonstrators and Observers exhibit an increase in c-Fos protein expression, as compared with their non-stressed peers (*p* < 0.01, Figure 3A). No such reaction was observed in BTBR mice. Moreover, upon stressing of the Demonstrators, the B6 mice (both the Demonstrators and Observers) displayed much higher expression of c-Fos protein than BTBR mice (both *p* < 0.01, Figure 3A). There was also an initial difference between non-stressed B6 and BTBR Observers, with the latter showing fewer c-Fos positive nuclei in the PrL region (*p* < 0.05, Figure 3A).

Very similar results were obtained for the infralimbic cortex (IL). Stressed B6 Demonstrators and Observers exhibited an increase of c-Fos expression, as compared to their non-stressed counterparts (*p* < 0.01 and *p* < 0.001 respectively, Figure 3B). Also, as in PrL, both the stressed B6 Demonstrators and Observers displayed higher numbers of c-Fos positive nuclei in the IL (*p* < 0.01, Figure 3B) and so did the non-stressed B6 Observers as compared with non-stressed BTBR Observers (*p* < 0.05, Figure 3B).

##### Amygdala

c-Fos expression in the amygdala resembled that in the prefrontal cortex.

In the basal/basolateral nucleus (BLA), exposure to stress in both Demonstrators (*p* < 0.05, Figure 3C) and Observers (*p* < 0.01, Figure 3C) of the B6 strain produced an increase in c-Fos protein expression, while in BTBR mice it did not. Both non-stressed and stressed BTBR Demonstrators (*p* < 0.05 and *p* < 0.01 respectively, Figure 3C) and Observers (both *p* < 0.01, Figure 3C) expressed less c-Fos in BLA than their B6 counterparts.

In the lateral nucleus (LA), stressed BTBR Demonstrators and Observers exhibited decreased c-Fos immunoreactivity as compared to non-stressed BTBR individuals (both *p* < 0.05, Figure 3D), while stressed Demonstrators and both non-stressed and stressed Observers of the B6 strain displayed higher c-Fos protein expression in LA than their BTBR counterparts (*p* < 0.05 and *p* < 0.01 respectively, Figure 3D).

In the lateral part of the central nucleus (CeL), stressed B6 Demonstrators showed higher c-Fos protein expression as compared to non-stressed B6 Demonstrators (*p* < 0.01, Figure 3E), while in the B6 Observers there was no such effect. In BTBR mice exposure to stress did not change c-Fos protein expression. Expression in stressed B6 Demonstrators was, however, higher than that in stressed BTBR Demonstrators (*p* < 0.01, Figure 3E). There was no difference in in c-Fos protein levels between Stressed Observers of the two mouse strains.

In the medial part of the central nucleus (CeM), stressed B6 Demonstrators exhibited an increase in the number of c-Fos positive nuclei as compared with non-stressed B6 Demonstrators (*p* < 0.05, Figure 3F) and stressed BTBR Demonstrators (*p* < 0.01, Figure 3F). Also stressed B6 Observers had more c-Fos positive nuclei in CeM than their BTBR counterparts (*p* < 0.05, Figure 3F).

In the medial nucleus (MeA) of the amygdala, stressed Demonstrators had higher c-Fos protein expression than non-stressed Demonstrators among B6 mice (*p* < 0.01, Figure 3G), but a contrary result was observed in BTBR mice (*p* < 0.05, Figure 3G). This made the difference in c-Fos protein expression between stressed Demonstrators of the two strains highly significant (*p* < 0.01, Figure 3G). The expression of c-Fos protein was also higher in B6 Observers (both non-stressed and stressed) as compared to BTBR Observers exposed to a similar treatment (*p* < 0.01, Figure 3G).

Similarly, in the cortical nucleus (CoA) of amygdala stressed BTBR Demonstrators had lower expression of c-Fos in CoA than their non-stressed counterparts (*p* < 0.05, Figure 3H): This expression was also lower than that observed in B6 stressed Demonstrators (*p* < 0.01 respectively, Figure 3H). Both non-stressed and stressed BTBR Observers had fewer c-Fos positive nuclei in CoA than their B6 counterparts (*p* < 0.01, Figure 3H).

##### Ventral hippocampus

In the CA1 field of the ventral hippocampus, B6 stressed Demonstrators experienced increased c-Fos protein expression as compared to their non-stressed counterparts (*p* < 0.05, Figure 3I). No such difference was observed for the B6 Observers. In BTBR Observers, on the other hand, a decrease in the number of c-Fos positive nuclei was observed upon exposure to a stressed cagemate (*p* < 0.05, Figure 3I). B6 stressed Demonstrators and both non-stressed and stressed Observers had higher c-Fos expression than their BTBR counterparts (*p* < 0.01, Figure 3I).

In the CA3 field of the ventral hippocampus, c-Fos protein expression was elevated in stressed B6 Observers as compared with their non-stressed counterparts (*p* < 0.05, Figure 3J). Stressed B6 Demonstrators and Observers had also more c-Fos positive nuclei in CA3 field than their BTBR counterparts (*p* < 0.01, Figure 3J).

c-Fos protein expression in the DG of the ventral hippocampus was higher in stressed B6 Demonstrators (*p* < 0.01, Figure 3K) and both non-stressed and stressed Observers (*p* < 0.01, Figure 3K) as compared to their BTBR counterparts. No stress dependent changes in the number of c-Fos positive nuclei were observed within either of the mouse strains.

### Experiment 2: Social Proximity

#### Behavioral Data

**Figure 4:**
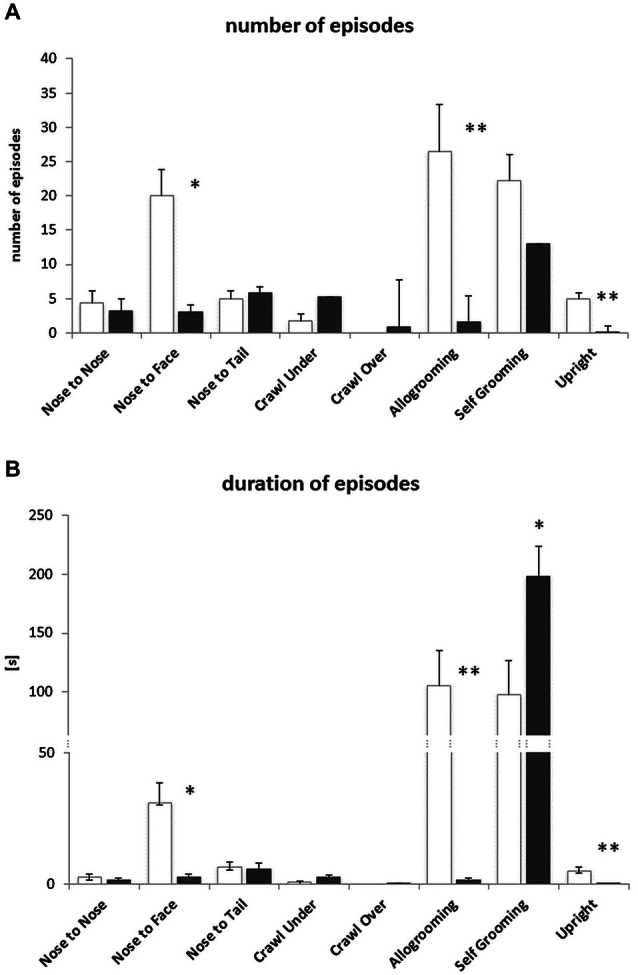
**Social Proximity test—BTBR *T^+^Itpr3^tf^/J* (BTBR) mice do not display prosocial behaviors towards c57BL/6J (B6) mice. (A)** Number of nose-to-nose, nose-to-face, nose-to-tail, crawl under, crawl over, allogrooming, selfgrooming and upright posture episodes, **(B)** the duration of these behaviors. White bars represent c57BL/6J (B6) mice, gray bars represent BTBR *T^+^Itpr3^tf^/J* (BTBR) mice. Values presented as mean. Error bars represent SEM. *p < 0.05 and **p < 0.01 for between strain comparisons.

During solitary exposure to the novel environment (the empty arena), BTBR mice groomed themselves more than the B6 mice (*p* < 0.01, data not shown). In the Social Proximity test, where social interactions were inevitable, the BTBR mice displayed a decreased number and amount of time spent on Nose-to-Face contacts, Allogrooming and the Upright postures (Figures 4A,B). The number of Selfgrooming bouts was slightly higher (n.s.) in B6 mice, but the bouts were significantly longer in BTBR mice (Figure 4B). The B6 mice also displayed a greater variety of other (non-social) types of behavior during the 10 min test, but the duration of these behaviors was similar in both mouse strains (data not shown).

#### c-Fos Protein Expression Data

The expression of c-Fos protein in control (Home Cage), solitary exposure to Novel Arena (Novel Arena) and Social Proximity (Social Proximity) conditions is summarized in Table 1.

**Table 1:** **c-Fos protein expression in brain structures of B6 and BTBR mice upon exposure to Home Cage, Novel Arena and Social Proximity**.

Structure	Home cage		Novel arena		Social proximity	
	B6	BTBR	B6	BTBR	B6	BTBR
**Cortex:**
Cingulate	0.17 ± 0.10	0.11 ± 0.04	0.35 ± 0.09	0.39 ± 0.14	0.18 ± 0.06	0.22 ± 0.04^#^
Prelimbic	0.16 ± 0.07	0.16 ± 0.08	0.37 ± 0.07^#^	0.42 ± 0.10	0.25 ± 0.05	0.41 ± 0.11^#^
Infralimbic	0.17 ± 0.07	0.11 ± 0.03	0.39 ± 0.06^#^	0.39 ± 0.06^#^	0.23 ± 0.07	0.41 ± 0.10^#^
**N. accumbens:**
Shell	0.07 ± 0.03	0.07 ± 0.04	0.17 ± 0.04	0.16 ± 0.04	0.24 ± 0.06^#^	0.19 ± 0.04^#^
Core	0.09 ± 0.03	0.10 ± 0.04	0.08 ± 0.02	0.10 ± 0.02	0.12 ± 0.03	0.11 ± 0.02
**Bed nucleus stria terminalis**	0.15 ± 0.03	0.11 ± 0.02	0.25 ± 0.04	0.19 ± 0.04	0.21 ± 0.04	0.24 ± 0.04^#^
**Amygdala:**
Basolateral	0.08 ± 0.01*	0.02 ± 0.01	0.17 ± 0.03^#^	0.09 ± 0.02^#^	0.16 ± 0.01^#^	0.11 ± 0.02^#^
Central	0.07 ± 0.01	0.03 ± 0.02	0.14 ± 0.02*^#^	0.08 ± 0.02	0.15 ± 0.03^#^	0.10 ± 0.02^#^
Medial	0.19 ± 0.03	0.12 ± 0.05	0.45 ± 0.08^#^	0.28 ± 0.06	0.43 ± 0.11^#^	0.41 ± 0.06^#^
Cortical	0.53 ± 0.07*	0.23 ± 0.06	0.78 ± 0.11	0.58 ± 0.09^#^	0.77 ± 0.11	0.85 ± 0.13^#^
**Hippocampus:**
CA1	0.14 ± 0.01	0.07 ± 0.02	0.23 ± 0.04	0.20 ± 0.07^#^	0.14 ± 0.02	0.16 ± 0.02^#^
CA2	0.12 ± 0.04	0.05 ± 0.00	0.19 ± 0.08	0.10 ± 0.02	0.09 ± 0.04	0.10 ± 0.03
CA3	0.19 ± 0.02	0.22 ± 0.08	0.31 ± 0.04^#^	0.37 ± 0.04	0.16 ± 0.03*^∧^	0.30 ± 0.04
Dentate gyrus	0.13 ± 0.02	0.14 ± 0.05	0.22 ± 0.02^#^	0.17 ± 0.05	0.14 ± 0.03^∧^	0.20 ± 0.04
**Hypothalamus:**
Paraventriclular n.	0.19 ± 0.04	0.15 ± 0.02	0.59 ± 0.10^#^	0.70 ± 0.13^#^	1.09 ± 0.09*^#^^∧^	0.54 ± 0.16
Dorsomedial n.	0.32 ± 0.02*	0.06 ± 0.01	0.45 ± 0.16	0.35 ± 0.10^#^	0.33 ± 0.05*	0.68 ± 0.09^#∧^
Ventromedial n.	0.14 ± 0.01*	0.03 ± 0.01	0.25 ± 0.05*	0.06 ± 0.00^#^	0.16 ± 0.06	0.05 ± 0.00^#^
V. Premammillary n.	0.28 ± 0.12	0.22 ± 0.09	0.37 ± 0.03	0.40 ± 0.09	0.25 ± 0.04*^∧^	0.62 ± 0.04^#∧^
**Periaqueductal gray:**
Dorsomedial c.	0.16 ± 0.06	0.08 ± 0.02	0.17 ± 0.07	0.21 ± 0.02^#^	0.10 ± 0.03	0.19 ± 0.04
Dorsolateral c.	0.16 ± 0.06	0.08 ± 0.01	0.18 ± 0.10	0.18 ± 0.01^#^	0.14 ± 0.01	0.14 ± 0.04
Lateral c.	0.19 ± 0.05	0.11 ± 0.03	0.25 ± 0.08	0.31 ± 0.05^#^	0.17 ± 0.04	0.29 ± 0.07
Ventrolateral c.	0.17 ± 0.03	0.10 ± 0.03	0.19 ± 0.04*	0.47 ± 0.10^#^	0.14 ± 0.02*	0.44 ± 0.12^#^
**Dorsal raphe n**.	0.24 ± 0.12	0.10 ± 0.04	0.16 ± 0.03*	0.30 ± 0.02^#^	0.24 ± 0.09	0.34 ± 0.09^#^

Values represent average ± SEM. *p < 0.05 for between strain comparisons, ^#^*p* < 0.05 for within strain comparisons with Home Cage group, ^∧^*p* < 0.05 for within strain comparisons between Novel Arena and Social Proximity conditions.

##### Cortex

Three distinct cortical regions of interest where analyzed: the cingulate (CG), prelimibic (PrL), and infralimbic (IL) cortices.

In the CG cortex no between-strain differences in any of the behavioral conditions tested (Home Cage, Novel Arena or Social Proximity exposure) were found. There was also no difference between these conditions in the B6 mouse strain. However, in BTBR mice an increase in c-Fos protein expression was significant after exposure to Social Proximity (as compared to Home Cage level, *p* < 0.05, Table 1).

Analysis performed for the PrL cortex also did not yield significant between-strain differences in any of the conditions. In B6 mice, however, exposure to Novel Arena produced an increase in the number of c-Fos positive nuclei (*p* = 0.05, Table 1). In BTBR mice, similar to the CG cortex, the increase in c-Fos protein expression was significant after Social Proximity exposure (*p* < 0.05, Table 1).

In the IL there were no between strain differences in any of the behavioral situations, but both B6 and BTBR mice showed an increase in c-Fos protein expression after exposure to Novel Arena as compared with Home Cage control (*p* < 0.05, Table 1). BTBR mice also showed an increase with Social Proximity (*p* < 0.05, Table 1).

##### Nucleus accumbens

The analysis of c-Fos protein expression in the NA was done separately for the nucleus accumbens shell (NAs) and core (NAc).

c-Fos protein expression in the shell region did not show any strain-dependent differences in any of the behavioral conditions tested. In both B6 and BTBR mice, c-Fos protein expression was elevated after exposure to Social Proximity as compared with Home Cage control (*p* < 0.05 for B6 and *p* = 0.05 for BTBR, Table 1).

The same analysis performed for the NAc region did not yield any significant differences with regard to either strain or testing condition.

##### Bed nucleus of stria terminalis

c-Fos protein expression in the bed nucleus of stria terminalis (BNST) did not reveal any strain-dependent differences. In BTBR mice, however, exposure to Social Proximity resulted in an increase in the number of c-Fos positive nuclei as compared with Home Cage control, (*p* < 0.05, Table 1).

##### Amygdala

The amygdala is a complex structure with as many as 13 different nuclei (Sah et al., 2003; Knapska et al., 2007). For the purpose of this analysis, we divided it into four major regions: the basolateral (BLA), central (CeA), medial (MeA) and cortical (CoA) nuclei.

Analysis of c-Fos protein expression in the basolateral nucleus revealed that baseline expression is higher in B6 mice than in BTBR mice (*p* < 0.05, Table 1). In both B6 and BTBR mice Novel Arena and Social Proximity exposure resulted in an increase in the number of c-Fos positive nuclei (as compared with Home Cage controls, *p* < 0.05 for B6 and *p* < 0.01 for BTBR, Table 1). No difference between Novel Arena and Social Proximity exposure was significant.

c-Fos protein expression in the central nucleus was found to be higher upon exposure to Novel Arena in B6 mice as compared with BTBR mice (*p* < 0.05, Table 1). The expression was elevated in B6 mice after exposure to both Novel Arena and Social Proximity (as compared to Home Cage controls, *p* < 0.05 and *p* = 0.05 respectively, Table 1), while in BTBR mice only exposure to the latter resulted in increased c-Fos protein expression (*p* < 0.05, Table 1).

c-Fos protein expression in the medial nucleus was similar in B6 and BTBR mice in all behavioral conditions. As with the CeA, in B6 mice expression was elevated after exposure to both Novel Arena and Social Proximity (*p* < 0.05, Table 1). BTBR mice only showed increased expression compared to Home Cage, on exposure to Social Proximity, (*p* < 0.05, Table 1).

In the cortical nucleus, baseline c-Fos protein expression in B6 mice was higher than that observed in BTBR mice (*p* < 0.05, Table 1). In B6 mice, exposure to either Social Proximity or to Novel Arena was not sufficient to induce elevated c-Fos protein expression. In BTBR mice both behavioral challenges evoked c-Fos elevation (*p* < 0.05 for Novel Arena and *p* < 0.01 for Social Proximity, Table 1).

##### Hippocampus

c-Fos protein was quantified in four regions of the dorsal hippocampus: CA1, CA2 and CA3 fields and the dentate gyrus (DG).

In the CA1 field, no strain-dependent differences in c-Fos protein expression were found. In B6 mice neither Novel Arena nor Social Proximity exposure evoked an increase in the number of c-Fos positive nuclei as compared with Home Cage controls. In BTBR mice, both conditions produced significant increases in c-Fos expression (*p* < 0.05, Table 1).

In the CA2 field, no strain or condition-related differences were observed.

In the CA3 field, BTBR mice showed higher expression of c-Fos protein after Social Proximity exposure than the B6 mice (*p* < 0.05, Table 1). In B6 mice, Novel Arena but not Social Proximity exposure produced a significant increase in the number of c-Fos positive nuclei as compared with Home Cage control (*p* < 0.05, Table 1). The difference in c-Fos levels between Novel Arena and Social Proximity exposure was significant for B6 mice (*p* < 0.05, Table 1). No significant increases in c-Fos expression were noted for Novel Arena or Social Proximity exposure in BTBR mice.

In the DG within strain comparisons for B6 mice indicated that Novel Arena exposure produced higher c-Fos expression in both Home Cage and Social Proximity (*p* < 0.05, Table 1). No differences were found for BTBR mice.

##### Hypothalamus

Four regions of the hypothalamus were chosen for the quantification of c-Fos protein expression: paraventricular (PVN), dorsomedial (DMH), ventromedial (VMH) and ventral premammillary nucleus (PMV).

In the paraventricular nucleus c-Fos was higher in B6 than in BTBR mice upon exposure to Social Proximity (*p* < 0.05, Table 1). In B6 mice both Novel Arena and Social Proximity exposures increased c-Fos protein expression above baseline (*p* < 0.05, Table 1). The increase evoked by Social Proximity was higher than that of the Novel Arena (*p* < 0.01, Table 1). In BTBR mice only exposure to Novel Arena induced a significant elevation in c-Fos protein expression as compared to Home Cage control (*p* < 0.05, Table 1).

In the dorsomedial nucleus, baseline c-Fos expression was higher for the B6 than for BTBR mice (*p* < 0.05, Table 1). In B6 mice, no significant condition-related differences in c-Fos expression were observed, whereas in BTBR mice both Novel Arena and Social Proximity exposure induced c-Fos expression higher than that of baseline (*p* < 0.05, Table 1). The c-Fos level observed after Social Proximity exposure in BTBR mice was also higher than that evoked by Novel Arena exposure (*p* < 0.05, Table 1).

c-Fos protein expression in the ventromedial nucleus was significantly lower in BTBR mice, both at baseline and after Novel Arena exposure as compared with B6 mice (*p* < 0.05, Table 1). Although Social Proximity scores for BTBR mice were also very low, the difference from B6 mice did not reach significance (*p* < 0.09). In B6 mice the differences between exposure groups were not significant, but in BTBR mice both Novel Arena and Social Proximity exposure produced an increase in the number of c-Fos positive nuclei as compared with Home Cage controls (*p* < 0.05, Table 1).

In the PMV, c-Fos protein expression was higher in BTBR than in B6 mice, after exposure to Social Proximity (*p* < 0.05, Table 1). In B6 mice only the difference between the levels of c-Fos protein evoked by Novel Arena and Social Proximity exposures was significant (*p* < 0.05, Table 1). In BTBR mice, c-Fos levels evoked by Social Proximity were higher than both the baseline and the Novel Arena levels (*p* < 0.05, Table 1).

##### Periaqueductal gray

For the purpose of c-Fos protein expression quantification the periaqueductal gray was divided into four columns: dorsomedial (DMPAG), dorsolateral (DLPAG), lateral (LPAG) and ventrolateral (VLPAG).

c-Fos expression in the dorsomedial column did not yield any significant differences for B6 vs. BTBR mice, nor were there significant exposure effects for B6 mice. In BTBR mice c-Fos positive nuclei increased with exposure to the Novel Arena (as compared to baseline, *p* < 0.05, Table 1), while the levels observed after Social Proximity exposure were not significantly different (*p* < 0.08).

The number of c-Fos positive nuclei was not different in the dorsolateral column for B6 and BTBR mice. In B6 mice, no changes in c-Fos levels were observed with exposure conditions. In BTBR mice, only the exposure to Novel Arena produced an increase in the number of c-Fos positive nuclei as compared with Home Cage control, (*p* < 0.05, Table 1).

In the lateral column, the only observed difference was an increase in c-Fos protein expression as compared with Home Cage control (*p* < 0.05, Table 1) in BTBR mice exposed to the Novel Arena.

In the ventrolateral column, c-Fos positive nuclei counts increased after exposure to either the Novel Arena or Social Proximity (*p* < 0.05, Table 1), but only in BTBR mice. The c-Fos protein expression in B6 mice in both of these conditions was lower than that observed in BTBR mice (*p* < 0.05, Table 1).

##### Dorsal raphe

c-Fos protein expression in the dorsal raphe nucleus was higher for BTBR than B6 mice after exposure to Novel Arena (*p* < 0.05, Table 1). BTBR mice showed more c-Fos positive nuclei in the dorsal raphe compared with Home Cage control after exposure to either Novel Arena or Social Proximity (*p* < 0.05, Table 1). No such effect was observed for the B6 mouse strain.

## Discussion

The current study confirms the asocial behavioral profile of the BTBR *T^+^Itpr3^tf^/J* (BTBR) mice and expands this profile by adding a new behavioral measure of social behavior impairment, the Transfer of Emotional Information test. It also allows, for the first time, to speculate about neuronal correlates of this impairment by comparing c-Fos protein expression profiles of BTBR and normo-social c57BL/6J (B6) mice.

### Experiment 1: The Transfer of Emotional Information Study

The behavioral response to the Transfer of Emotional Information from a stressed conspecific clearly confirms the notion presented by Wöhr (2015), that *BTBR mice are capable of detecting change in the social context of the environment (here the changed emotional status of the cagemate), but that their response to it is altered*. Unlike B6 mice, BTBR males do not increase the number and duration of contacts and the episodes of following of the stressed Demonstrators. Instead they seem to withdraw from (already low levels of) nose-to-nose and nose-to tail interactions. The relatively high (comparable to B6 mice) number of total contacts made by the unstressed BTBR mice, reflects a high number of brief side touches resulting from avoidance of nose-to-nose contacts. Their duration, however, is much shorter than for B6 mice. This is accompanied by a lack of increase, and even a decrease, in c-Fos protein expression in many of the brain structures relevant for emotional contagion in mice.

**Figure 5:**
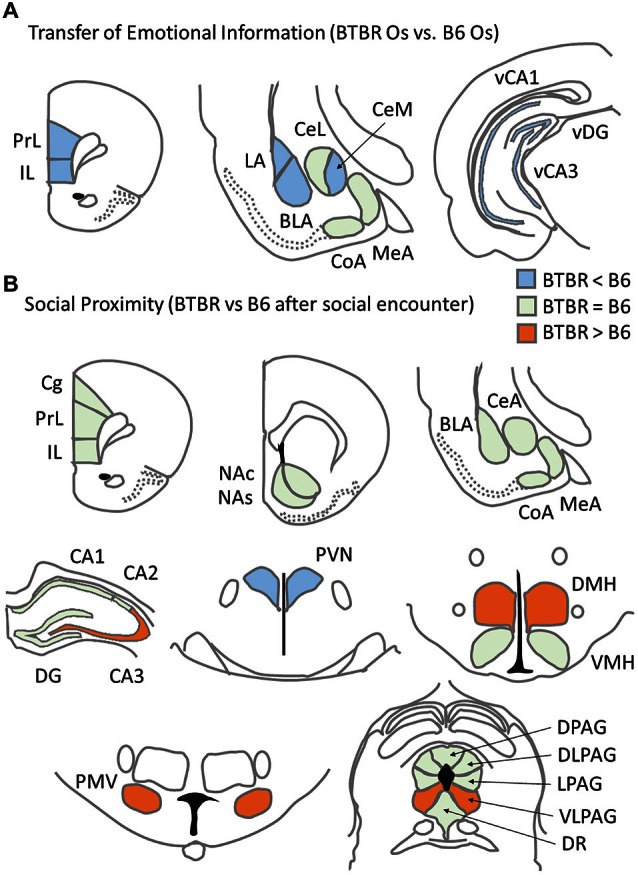
**Summary of c-Fos protein expression patterns related to asocial behavior for: (A) c57BL/6J (B6) mice and BTBR *T^+^Itpr3^tf^/J* (BTBR) mice Observers exposed to a stressed cagemate in the Transfer of Emotional Information test and **(B)** B6 and BTBR mice exposed to a social stimulus (unfamiliar B6 male) during Social proximity test**.

In the Transfer of Emotional Information test, stressed B6 Demonstrator mice showed increased c-Fos expression in the medial prefrontal cortex (both PrL and IL parts), the amygdala (basolateral, central medial, central lateral, and medial nuclei) and the CA1 field of the ventral hippocampus. B6 Observers exposed to a stressed Demonstrator also showed increases in the number of c-Fos positive nuclei in the PrL and IL medial prefrontal cortex, basolateral nucleus of amygdala and the CA3 field of the ventral hippocampus. In contrast, the exposure to neither direct stress (experienced by the Demonstrators) nor remote stress (experienced by the Observers) produced an increase in c-Fos protein expression in any of the brain regions analyzed in the BTBR mice (Figure 5A). Instead, such behavioral challenge decreased c-Fos protein expression in the lateral, medial, and cortical nuclei of the amygdala and in the CA1 field and the DG of the ventral hippocampus in BTBR Demonstrators as well as in the CA1 field of the ventral hippocampus in BTBR Observers. Whether lower c-Fos expression in these amygdalar and hippocampal structures in stressed BTBR Demonstrators is linked to their inability to learn the context associated with aversive, unconditioned stimuli during fear conditioning (MacPherson et al., 2008; Scattoni et al., 2013; Stapley et al., 2013) is an intriguing possibility. In sum, *the comparison of results for the two strains indicates that BTBR mice showed a widespread decrease, opposite to that observed in the B6 strain, of c-Fos protein expression during direct or transferred stress*.

The pattern of increases in c-Fos protein expression in the amygdala of B6 mice seems to be species-specific. When compared with c-Fos protein expression patterns of rats tested in the same experimental paradigm (Knapska et al., 2006), limited similarities can be found for the Demonstrators. Increased c-Fos protein levels were found in the rat basolateral, lateral and medial nuclei of the amygdala. The present findings of B6 c-Fos increases in the basolateral and medial (as well as central lateral and central medial) nuclei, but not in the LA, provide a substantial but incomplete parallel to the rat pattern. In the rat Observers, c-Fos protein expression was higher than in the Demonstrators in the central nucleus of amygdala. This does not hold true for B6 mice. In B6 mice, however, there was a significant increase in the number of c-Fos positive nuclei in the central lateral and central medial amygdala regions in the stressed Demonstrators, an effect not present in their rat counterparts. The increase in the number of c-Fos positive nuclei in B6 Observers exposed to a stressed cagemate was observed only for the basolateral nucleus of amygdala, while in rats it was true for the basolateral, lateral and medial nuclei. While Knapska et al. (2006) focused on neuronal activation patterns in the amygdala alone, the latest study from the same group (Mikosz et al., in press) also examined c-Fos protein expression in the medial prefrontal cortex and reported increases in the number of c-Fos positive nuclei therein in both the Demonstrators and the Observers upon undergoing a stressful experience. In the current study, a similar phenomenon was observed for B6 mice, but not BTBR mice.

The species-specificity of these patterns of c-Fos protein expression in Observers may reflect different strategies of fear transmission employed by rats and mice. While scent marking is a particularly important mode of communication in mice (Arakawa et al., 2008), rats rely more than mice on ultrasonic vocalizations (Brudzynski, 2013). The latter difference was clearly marked during the Transfer of Emotional Information test, where in rats many 50 kHz calls were recorded during the reunion of the Demonstrators with the Observers (Rokosz et al., in preparation), while very little or no such vocalization was recorded in the current mouse study. Fear responses following exposure to threatening predators or conspecifics involve the medial nucleus of amygdala (Canteras et al., 2012) while olfactorially-mediated fear transmission from mother to infant rats involves the basolateral nucleus (Debiec and Sullivan, 2014), suggesting that the hyper activation of these areas observed in B6 Demonstrator mice, and of the basolateral nucleus alone in the Observers, may reflect the more comprehensive role of olfaction in this species.

### Experiment 2: The Social Proximity Test

Since, as reported by Defensor et al. (2011), BTBR pairs of mice display substantially reduced interactions in the Social Proximity test, the neuronal activation pattern evoked by such exposure is not expected to be comparable to the pattern evoked by a period of intense social activity experienced by B6-B6 pairs. In order to provoke any social interaction with the BTBR test animals we employed unfamiliar B6 males as partners for either B6 or BTBR test animals to study c-Fos protein expression evoked by brief, 10 min periods of social interaction. Although paired with a highly social B6 mouse, BTBR still showed reduced interactions with the partner, in line with the data presented by Defensor et al. (2011). We also confirmed that BTBR mice show reduced grooming of their partner (low number and duration of allogrooming), but perform long bouts of selfgrooming (seen also in semi-natural environment, Pobbe et al., 2010).

This asocial behavior was paralleled by distinct c-Fos protein expression patterns found in B6 and BTBR mice upon exposure to Social Proximity (Figure 5B). Contrary to the observations from Experiment 1, *broader activation (understood as significantly higher c-Fos protein expression after Social proximity exposure as compared to Home Cage condition) was observed in BTBR mice* (15 brain regions) than in B6 mice (five brain regions). The c-Fos protein level was *higher* in BTBR mice as compared with B6 mice in four brain regions: the *CA3 field of the hippocampus, dorsomedial and ventral premammillary hypothalamic nuclei* and the ventrolateral column of the periaqueductal gray. The number of c-Fos positive nuclei in the *paraventricular nucleus of the hypothalamus*, however, *was found higher in the B6 mice*.

The functional significance of these findings is supported by data showing that repeated social defeat produces structural alterations of the apical or basal (or both) dendrites of hippocampal CA3 pyramidal neurons, along with changes in hippocampal LTP or LTD (Buwalda et al., 2005). Since hippocampal projections are involved in negative feedback of the hypothalamic-pituitary-adrenal (HPA) axis (Jacobson and Sapolsky, 1991), the observed increased activation of CA3 region may be linked with decreased activity of the PVN in BTBR mice. The PVN it is one of the two main brain areas that release oxytocin in response to affiliative conspecific stimuli (Dabrowska et al., 2011). Its activation in B6 mice may be related to high social preference presented by these mice (Pearson et al., 2012). The lack of such activation in BTBR mice could indicate that these mice do not find social contacts rewarding. The decreased activation of PVN in BTBR mice could also be a result of the up-regulation of baseline HPA function (Benno et al., 2009; Frye and Llaneza, 2010; Gould et al., 2014) and a formation of a ceiling effect for the c-Fos protein response to social challenge. The enhanced c-Fos protein expression in the DMH and PMV of the BTBR mice, on the other hand, is in line with studies showing that these structures belong to the corticoliberin (CRH) pathway (Bernardis and Bellinger, 1998; Henry et al., 2005) and are responsible for regulation of heart rate and blood pressure in response to stress. While reversible inactivation of DMH has a panicolytic effect of reducing flight in an elevated T-maze (Nascimento et al., 2010), the increased DMH c-Fos protein expression to social stimuli in BTBR mice may reflect the active avoidance of contact in the confined small space of the Social Proximity chamber presented by these mice. The PMV, together with the medial nucleus of the amygdala, is activated by conspecific odor stimulation (Donato et al., 2010; Kim et al., 2015). Roullet et al. (2011) showed that B6 mice produce more scent marks in response to BTBR mice than to B6 males. Such stronger olfactory stimulation could have provoked higher c-Fos protein expression in the PMV of BTBR mice.

Although not specific for social challenge, the consistently high levels of c-Fos protein expression to either Novel arena or Social proximity in the ventrolateral column of the periaqueductal gray (VLPAG) of BTBR mice are also noteworthy. As a structure responsible for defensive freezing, VLPAG usually responds to highly aversive threats (Koutsikou et al., 2014). Its activation in BTBR mice points to a high stress level of these animals and a possible panic-like reaction to both challenges.

### Conclusion

The current study focused on describing the neurobiological background of anti-social behaviors observed in the BTBR mouse model of idiopathic ASD. The emerging view is that BTBR mice have a distinct pattern of neuronal response to socially aversive contexts than the normo-social B6 mice. While between-subject transfer of aversive information inhibits c-Fos mediated transcription activation in the structures responsible for social learning in BTBR mice, social interaction in close proximity activates transcription in more regions in BTBR mice than in B6 mice. Analysis of strain-specific areas in which the Social Proximity stimulus selectively enhances c-Fos expression for BTBR mice yielded a relatively small group: the CA3 of the hippocampus, along with the dorsomedial and ventral premammillary nuclei in the hypothalamus, leaving the paraventricluar nucleus of the hypothalamus hypoactive. These particular areas overlap substantially with those of earlier studies reporting social stress effects on regional brain activation. Moreover, BTBR mice react with higher activation of the ventrolateral column of the periaqueductal gray to both unwanted social contact and novel environment exposure. This indicates that BTBR mice perceive these challenges as more aversive. This is in line with anecdotal reports of panic reactions of autistic patients faced with novel social situations. The data gathered here strongly support the notion that BTBR mice, unlike B6 mice, react inadequately to social stressors and do not display simple forms of empathy. With a high resemblance to the lack of empathy displayed by autistic patients, this further validates the BTBR mouse model of idiopathic autism.

## Conflict of Interest Statement

The authors declare that the research was conducted in the absence of any commercial or financial relationships that could be construed as a potential conflict of interest.

## Footnotes

^1^http://pmbogusz.net/

## References

[B1] American Psychiatric Association (2013). Diagnostic and Statistical Manual of Mental Disorders. 5th Edn. Washington, DC: American Psychiatric Publishing.

[B2] Arakawa H. Blanchard D. C. Arakawa K. Dunlap C. Blanchard R. J. (2008). Scent marking behavior as an odorant communication in mice. Neurosci. Biobehav. Rev. 32, 1236–1248. 10.1016/j.neubiorev.2008.05.012 18565582 PMC2577770

[B3] Benno R. Smirnova Y. Vera S. Liggett A. Schanz N. (2009). Exaggerated responses to stress in the BTBR T+tf/J mouse: an unusual behavioral phenotype. Behav. Brain Res. 197, 462–465. 10.1016/j.bbr.2008.09.041 18977396

[B4] Bernardis L. L. Bellinger L. L. (1998). The dorsomedial hypothalamic nucleus revisited: 1998 update. Proc. Soc. Exp. Biol. Med. 218, 284–306. 10.3181/00379727-218-44296 9714072

[B5] Betancur C. (2011). Etiological heterogeneity in autism spectrum disorders: more than 100 genetic and genomic disorders and still counting. Brain Res. 1380, 42–77. 10.1016/j.brainres.2010.11.078 21129364

[B6] Blanchard D. C. Defensor E. B. Meyza K. Z. Pobbe R. L. Pearson B. L. Bolivar V. J.. (2012). BTBR T+tf/J mice: autism-relevant behaviors and reduced fractone-associated heparan sulfate. Neurosci. Biobehav. Rev. 36, 285–296. 10.1016/j.neubiorev.2011.06.008 21741402 PMC3208071

[B7] Bredy T. W. Barad M. (2008). Social modulation of associative fear learning by pheromone communication. Learn. Mem. 16, 12–18. 10.1101/lm.1226009 19117912 PMC2632855

[B8] Brudzynski S. M. (2013). Ethotransmission: communication of emotional states through ultrasonic vocalization in rats. Curr. Opin. Neurobiol. 23, 310–317. 10.1016/j.conb.2013.01.014 23375168

[B9] Buwalda B. Kole M. H. Veenema A. H. Huininga M. de Boer S. F. Korte S. M.. (2005). Long-term effects of social stress on brain and behavior: a focus on hippocampal functioning. Neurosci. Biobehav. Rev. 29, 83–97. 10.1016/j.neubiorev.2004.05.005 15652257

[B10] Canteras N. S. Mota-Ortiz S. R. Motta S. C. (2012). What ethologically based models have taught us about the neural systems underlying fear and anxiety. Braz. J. Med. Biol. Res. 45, 321–327. 10.1590/s0100-879x2012007500042 22450374 PMC3854166

[B11] Cassidy S. Mitchell P. Chapman P. Ropar D. (2015). Processing of spontaneous emotional responses in adolescents and adults with autism spectrum disorders: effect of stimulus type. Autism Res. [Epub ahead of print]. 10.1002/aur.1468 25735657 PMC4964927

[B12] Chen Q. Panksepp J. B. Lahvis G. P. (2009). Empathy is moderated by genetic background in mice. PLoS One 4:e4387. 10.1371/journal.pone.0004387 19209221 PMC2633046

[B13] Dabrowska J. Hazra R. Ahern T. H. Guo J. D. McDonald A. J. Mascagni F.. (2011). Neuroanatomical evidence for reciprocal regulation of the corticotrophin-releasing factor and oxytocin systems in the hypothalamus and the bed nucleus of the stria terminalis of the rat: implications for balancing stress and affect. Psychoneuroendocrinology 36, 1312–1326. 10.1016/j.psyneuen.2011.03.003 21481539 PMC3142325

[B14] Debiec J. Sullivan R. M. (2014). Intergenerational transmission of emotional trauma through amygdala-dependent mother-to-infant transfer of specific fear. Proc. Natl. Acad. Sci. U S A 111, 12222–12227. 10.1073/pnas.1316740111 25071168 PMC4142995

[B15] Defensor E. B. Pearson B. L. Pobbe R. L. H. Bolivar V. J. Blanchard D. C. Blanchard R. J. (2011). A novel social proximity test suggests patterns of social avoidance and gaze aversion-like behavior in BTBR T+ tf/J mice. Behav. Brain Res. 217, 302–308. 10.1016/j.bbr.2010.10.033 21055421 PMC3124342

[B16] de Waal F. B. (2008). Putting the altruism back into altruism: the evolution of empathy. Annu. Rev. Psychol. 59, 279–300. 10.1146/annurev.psych.59.103006.093625 17550343

[B17] Donato J. Jr. Cavalcante J. C. Silva R. J. Teixeira A. S. Bittencourt J. C. Elias C. F. (2010). Male and female odors induce Fos expression in chemically defined neuronal population. Physiol. Behav. 99, 67–77. 10.1016/j.physbeh.2009.10.012 19857504

[B18] Felix-Ortiz A. C. Tye K. M. (2014). Amygdala inputs to the ventral hippocampus bidirectionally modulate social behavior. J. Neurosci. 34, 586–595. 10.1523/JNEUROSCI.4257-13.2014 24403157 PMC3870937

[B19] Fombonne E. (2003). Epidemiological surveys of autism and other pervasive developmental disorders: an update. J. Autism Dev. Disord. 33, 365–382. 10.1023/A:1025054610557 12959416

[B20] Frye C. A. Llaneza D. C. (2010). Corticosteroid and neurosteroid dysregulation in an animal model of autism, BTBR mice. Physiol. Behav. 100, 264–267. 10.1016/j.physbeh.2010.03.005 20298706 PMC2860004

[B21] Geschwind D. H. (2011). Genetics of autism spectrum disorders. Trends Cogn. Sci. 15, 409–416. 10.1016/j.tics.2011.07.003 21855394 PMC3691066

[B22] Gould G. G. Burke T. F. Osorio M. D. Smolik C. M. Zhang W. Q. Onaivi E. S.. (2014). Enhanced novelty-induced corticosterone spike and upregulated serotonin 5-HT1A and cannabinoid CB1 receptors in adolescent BTBR mice. Psychoneuroendocrinology 39, 158–169. 10.1016/j.psyneuen.2013.09.003 24126181 PMC3893037

[B23] Hatfield E. Rapson R. L. Le Y. L. (2009). “Emotional contagion and empathy,” in The social neuroscience of empathy, eds Decety J. Ickes W. (Boston, MA: MIT Press), 19–30.

[B24] Henry B. A. Lightman S. L. Lowry C. A. (2005). Distribution of corticotropin-releasing factor binding protein-immunoreactivity in the rat hypothalamus: association with corticotropin-releasing factor-, urocortin 1- and vimentin-immunoreactive fibers. J. Neuroendocrinol. 17, 135–144. 10.1111/j.1365-2826.2005.01274.x 15796765

[B25] Jacobson L. Sapolsky R. (1991). The role of the hippocampus in feedback regulation of the hypothalamic-pituitary-adrenocortical axis. Endocr. Rev. 12, 118–134. 10.1210/edrv-12-2-118 2070776

[B26] Jeon D. Kim S. Chetana M. Jo D. Ruley H. E. Lin S. Y.. (2010). Observational fear learning involves affective pain system and Cav1.2 Ca2+ channels in ACC. Nat. Neurosci. 13, 482–488. 10.1038/nn.2504 20190743 PMC2958925

[B27] Jo H. Schieve L. A. Rice C. E. Yeargin-Allsopp M. Tian L. H. Blumberg S. J.. (2015). Age at Autism Spectrum Disorder (ASD) diagnosis by race, ethnicity and primary household language among children with special health care needs, United States, 2009–2010. Matern. Child Health J. [Epub ahead of print]. 10.1007/s10995-015-1683-4 25701197 PMC4500845

[B28] Kim Y. Venkataraju K. U. Pradhan K. Mende C. Taranda J. Turanga S. C.. (2015). Mapping social behavior induced brain activation at cellular resolution in the mouse. Cell Rep. 10, 292–305. 10.1016/j.celrep.2014.12.014 25558063 PMC4294964

[B29] Knapska E. Mikosz M. Werka T. Maren S. (2009). Social modulation of learning in rats. Learn. Mem. 17, 35–42. 10.1101/lm.1670910 20042480 PMC3960044

[B480] Knapska E. Radwanska K. Werka T. Kaczmarek L. (2007). Functional internal complexity of amygdala: focus on gene activity mapping after behavioral training and drugs of abuse. Physiol. Rev. 87, 1113–1173. 10.1152/physrev.00037.2006 17928582

[B30] Knapska E. Nikolaev E. Boguszewski P. Walasek G. Blaszczyk J. Kaczmarek L.. (2006). Between-subject transfer of emotional information evokes specific pattern of amygdala activation. Proc. Natl. Acad. Sci. U S A 103, 3858–3862. 10.1073/pnas.0511302103 16497832 PMC1533786

[B31] Koutsikou S. Crook J. J. Earl E. V. Leith J. L. Watson T. C. Lumb B. M.. (2014). Neural substrates underlying fear-evoked freezing: the periaqueductal gray-cerebellar link. J. Physiol. 592(Pt. 10), 2197–2213. 10.1113/jphysiol.2013.268714 24639484 PMC4027863

[B32] MacPherson P. McGaffigan R. Wahlsten D. Nguyen P. V. (2008). Impaired fear memory, altered object memory and modified hippocampal synaptic plasticity in split-brain mice. Brain Res. 1210, 179–188. 10.1016/j.brainres.2008.03.008 18417102

[B33] Maenner M. J. Durkin M. S. (2010). Trends in the prevalence of autism on the basis of special education data. Pediatrics 126, e1018–e1025. 10.1542/peds.2010-1023 20974790

[B34] Meyza K. Z. Defensor E. B. Jensen A. L. Corley M. J. Pearson B. L. Pobbe R. L.. (2013). The BTBR T+ tf/J mouse model for autism spectrum disorders-in search of biomarkers. Behav. Brain Res. 251, 25–34. 10.1016/j.bbr.2012.07.021 22958973 PMC3529977

[B49] Mikosz M. Nowak A. Werka T. Knapska E. (in press). Sex differences in social modulation of learning in rats. 10.1038/srep18114 PMC4677340 26655917

[B35] Nascimento J. O. Zangrossi H. Jr. Viana M. B. (2010). Effects of reversible inactivation of the dorsomedial hypothalamus on panic- and anxiety-related responses in rats. Braz. J. Med. Biol. Res. 43, 869–873. 10.1590/s0100-879x2010007500075 20694443

[B36] Panksepp J. B. Lahvis G. P. (2011). Rodent empathy and affective neuroscience. Neurosci. Biobehav. Rev. 35, 1864–1875. 10.1016/j.neubiorev.2011.05.013 21672550 PMC3183383

[B37] Panksepp J. Panksepp J. B. (2013). Toward a cross-species understanding of empathy. Trends Neurosci. 36, 489–496. 10.1016/j.tins.2013.04.009 23746460 PMC3839944

[B38] Paxinos G. Franklin K. B. J. (2001). The Mouse Brain in Stereotaxic Coordinates. 2nd Edn. San Diego: Academic Press.

[B39] Pearson B. L. Bettis J. K. Meyza K. Z. Yamamoto L. Y. Blanchard D. C. Blanchard R. J. (2012). Absence of social conditioned place preference in BTBR T+tf/J mice: relevance for social motivation testing in rodent models of autism. Behav. Brain Res. 233, 99–104. 10.1016/j.bbr.2012.04.040 22562042 PMC3378798

[B40] Pobbe R. L. Pearson B. L. Defensor E. B. Bolivar V. J. Blanchard D. C. Blanchard R. J. (2010). Expression of social behaviors of C57BL/6J versus BTBR inbred mouse strains in the visible burrow system. Behav. Brain Res. 214, 443–449. 10.1016/j.bbr.2010.06.025 20600340 PMC2928226

[B41] Roullet F. I. Wöhr M. Crawley J. N. (2011). Female urine-induced male mice ultrasonic vocalizations, but not scent-marking, is modulated by social experience. Behav. Brain Res. 216, 19–28. 10.1016/j.bbr.2010.06.004 20540967 PMC3094925

[B51] Sah P. Faber E. S. Lopez De Armentia M. Power J. (2003). The amygdaloid complex: anatomy and physiology. Physiol. Rev. 83, 803–834. 10.1152/physrev.00002.2003 12843409

[B42] Scattoni M. L. Gandhy S. U. Ricceri L. Crawley J. N. (2008). Unusual repertoire of vocalizations in the BTBR T+tf/J mouse model of autism. PLoS One 3:e3067. 10.1371/journal.pone.0003067 18728777 PMC2516927

[B43] Scattoni M. L. Martire A. Cartocci G. Ferrante A. Ricceri L. (2013). Reduced social interaction, behavioral flexibility and BDNF signalling in the BTBR T+ tf/J strain, a mouse model of autism. Behav. Brain Res. 251, 35–40. 10.1016/j.bbr.2012.12.028 23270976

[B44] Scattoni M. L. Ricceri L. Crawley J. N. (2011). Unusual repertoire of vocalizations in adult BTBR T+tf/J mice during three types of social encounters. Genes Brain Behav. 10, 44–56. 10.1111/j.1601-183x.2010.00623.x 20618443 PMC2972364

[B45] Stapley N. W. Guariglia S. R. Chadman K. K. (2013). Cued and contextual fear conditioning in BTBR mice is improved with training or atomoxetine. Neurosci. Lett. 549, 120–124. 10.1016/j.neulet.2013.06.032 23827222

[B46] Wöhr M. (2015). Effect of social odor context on the emission of isolation-induced ultrasonic vocalizations in the BTBR T+tf/J mouse model for autism. Front. Neurosci. 9:73. 10.3389/fnins.2015.00073 25852455 PMC4364166

[B47] Wöhr M. Roullet F. I. Crawley J. N. (2011). Reduced scent marking and ultrasonic vocalizations in the BTBR T+tf/J mouse model of autism. Genes Brain Behav. 10, 35–43. 10.1111/j.1601-183x.2010.00582.x 20345893 PMC2903641

[B48] Yang M. Abrams D. N. Zhang J. Y. Weber M. D. Katz A. M. Clarke A. M.. (2013). Low sociability in BTBR *T+tf/J* mice is independent of partner strain. Physiol. Behav. 107, 649–662. 10.1016/j.physbeh.2011.12.025 22245067 PMC3330157

